# Explanatory Model of Perceived Stress in the General Population: A Cross-Sectional Study in Peru During the COVID-19 Context

**DOI:** 10.3389/fpsyg.2021.673945

**Published:** 2021-06-23

**Authors:** Alicia Boluarte-Carbajal, Alba Navarro-Flores, David Villarreal-Zegarra

**Affiliations:** ^1^Facultad de Ciencias de la Salud, Universidad César Vallejo – Lima Norte, Lima, Peru; ^2^Instituto Peruano de Orientación Psicológica, Lima, Peru; ^3^Facultad de Medicina, Universidad Nacional Federico Villarreal, Lima, Peru

**Keywords:** COVID-19, perceived stress, general population, Peru, structural equation modeling

## Abstract

**Background:**

The COVID-19 pandemic had negatively impact mental health worldwide. High prevalence of stress had been previously reported in populations during this context. Many theoretical frameworks had been proposed for explaining the stress process, we aim to proposed and explanatory model for the genesis of perceived stress in Peruvian general population.

**Method:**

We conducted an online survey in Peruvian general population assessing sociodemographic variables and evaluating mental health conditions by using The Perceived Stress Scale (PSS-10), Positive Affect and Negative Affect Scale (PANAS), Generalized Anxiety Disorder scale (GAD-7), Patient Health Questionnaire (PHQ-9), and a numerical rating scale (NRS) for fear of COVID-19. Correlation analysis was conducted for the variables of interest. Two regression models were constructed to explore related factor to the dimensions of perceived stress. Finally, a structural regression model was performed with the independent variables.

**Results:**

Data of 210 individuals was analyzed. Ages ranged from 15 to 74 years and 39% were women. Additionally, 65.2% of the participants had at least one mental health conditions (depression, anxiety, or stress symptoms). Perceived self-efficacy and positive affect (PA) were correlated, as perceived helplessness with anxious symptoms and negative affect (NA). Regression analysis showed that sex, anxiety symptoms, and NA explained perceived helplessness while positive and NA explained self-efficacy. The structural regression model analysis identified that fear of COVID-19 (composed of fear of infecting others and fear of contagion), predicted mental health conditions (i.e., depressive or anxiety symptoms); also, mental health conditions were predicted by PA and NA. Perceived helplessness and Perceived self-efficacy were interrelated and represented the perceived stress variable.

**Conclusion:**

We proposed an explanatory model of perceived stress based on two correlated dimensions (self-efficacy and helplessness) in the Peruvian general population during the context of the COVID-19 pandemic, with two out of three individuals surveyed having at least one mental health condition.

## Introduction

In the year 2020, the global social, economic, and health structures were redefined by the challenging context of a pandemic. Sequentially since March 2020, when the SARS-Cov-2 infection was declared the COVID-19 pandemic (Organization, 2020), governments around the globe set strict rules of social restrictions. By mid-April, most of the countries in the world were under some kind of confinement (Hale et al., 2021), representing a unique setting for behavior and psychology research (Bates et al., 2020).

In Peru, the first case of COVID-19 was diagnosed on March 8, 2020 and a national lockdown was installed as soon as March 16. Despite this early response, the disease spread around the country rapidly and reached the worst metrics for pandemic control worldwide by August (University, 2020). These outcomes were poorly predicted by epidemiological models (Pacheco-Barrios et al., 2020). Some potential related factors are socioeconomic inequities, high rate of informal business, difficulties to the access of supplies (Herrera Romero and Reys, 2020) and health services (Nevin et al., 2019), on the basis of a fragile and fragmented health care system (World Health Organization, 2003; Sánchez-Moreno, 2014). The governmental Peruvian response for mental health preservation during COVID-19 had been insufficient too and the technical guidelines proposed were logistically unrealistic in terms of implementation (Giraldo, 2020).

This situation as unprecedented, could be compared with other negative environmental contexts, such natural disasters in which mental health outcomes are impaired (Stough and North, 2018). In addition to mandatory social restrictions, other consequences of the pandemic as dealing with the disease as a patient, the fear of getting infected or to infect others, grieving with human losses, economic difficulties (i.e., unemployment, increase of debts, poor access to food, and primary-need supplies, etc.) and feeling uncertain about the future had been proposed as important stressors related to this context (Hagger et al., 2020).

Accordingly, systematic reviews about the impact of the COVID-19 pandemic in mental health reported high frequencies of depression (21.94–33.7%), anxiety (13.29–31.9%), and stress (13.29–29.6%) (Salari et al., 2020; Cénat et al., 2021). According to another meta-analysis the overall prevalence of psychological distress during COVID-19 pandemic rose to 41.1%, being higher in patients with suspicion of infection (99.6%) when compared to the general population (31.1%) (Wu et al., 2021). Additionally, according to a survey using the COVISTRESS questionnaire, assessed in 67 countries of the five continents, the self-reported symptoms of depression, anxiety and stress increased by 21.62, 16.71, and 21.8%, respectively (Ugbolue et al., 2020). In order to evaluate symptoms of mental health impairment, generic scales like the 9-item Patient Health Questionnaire (PHQ-9) (Levis et al., 2019) and the 7-item Generalized Anxiety Disorder (GAD-7) (Toussaint et al., 2020) had been broadly accepted for the appropriate screening measures. Many new scales design specifically for the current pandemic had been developed situation (Bernardo et al., 2020; Lee, 2020; Tavormina et al., 2020), however, their lack of validation in our context. Still, the generic scales are being used in the pandemic context (Luan et al., 2020), and standardized a point for comparison with other populations.

According to Cohen’s original theory of perceived stress, the stressor is not the potentially omnipresent life event that occurs to the individual, but rather “the cognitively mediated emotional response to the target event” (Cohen et al., 1983). Therefore, when evaluating perceived stress, we are scoring a global response that depends on various personal traits such as coping mechanisms, baseline psychopathological state or personality types. However, perceived stress is a complex concept that depends on factors such as the perception of how self-effective the person is in coping with demands from the external environment and the perception of helplessness as an internal response to negative emotions and lack of control facing stress (Liu et al., 2020). Different studies consider that the distinction between both dimensions represent separate components of the stress experience, so they should not be included in a single construct (Baik et al., 2019; Liu et al., 2020). Therefore, we consider that the perceived stress response can be understood only from these two separate variables.

**FIGURE 1:**
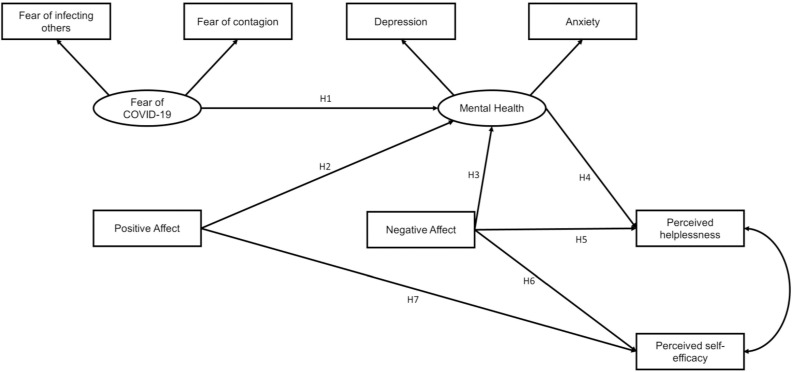
Proposed model that explains the two dimensions of perceived stress.

The present study investigated the potential factors that explain the two dimensions of perceived stress of COVID-19 infection among the general Peruvian population. The following hypotheses were formulated based on the literature mentioned above (see Figure 1): H1: fear of COVID-19 is positively associated with mental health problems such as anxiety and depression; H2: PA is negatively associated with the presence of mental health problems such as anxiety and depression; H3: NA is positively associated with the presence of mental health problems such as anxiety and depression; H4: mental health problems are positively associated with the perceived helplessness component of stress; H5: NA is positively associated with the perceived helplessness component of stress; H6: NA is positively associated with the perceived self-efficacy component of stress; H7: PA is negatively associated with the perceived self-efficacy component of stress.

## Materials and Methods

### Study Design

This research is of an empirical nature since it aims to address an specific problem, to which a response is sought following a particular strategy (Ato and López-García, 2013). The strategy is associative, where the functional relationship between various variables (sociodemographic factors and psychological variables) is explored. The design of the study was explanatory since it seeks to identify a causal relationship between the variables.

### Setting

This study was conducted online in the general population of Peru. Four main sources of stress during COVID-19 had been proposed (Biondi and Iannitelli, 2020), some examples according to our setting are described following:

a) Pandemic-related: the advancement of disease propagation, the nature of the disease transmission since air-borne epidemics had been related to higher stress outcomes (Luo et al., 2020), the absence of specific treatment, the lack of a vaccine and the uncertainty of dealing with a novel virus could be some examples.
b) Information-related: as the misinformation and panic generated by the media (“Infodemics”) (The Lancet Infectious Diseases, 2020). In Peru, the massive spread of non-evidence-based treatments sometimes endorsed by governmental entities had an impact on general population decision of massive off-label self-medication and consequent shortage of drugs needed for the original prescriptions (Alvarez-Risco et al., 2020).
c) Lockdown-related: Not only about social isolation but the prohibition of certain activities that were not of “primary need,” like social reunions, concerts, tourism, art exhibitions, etc. (Jurblum et al., 2020; Yamamoto et al., 2020). It was reported that during lockdown individuals perceived time as moving slower, and this perceptual change of having more free time was related to higher levels of stress and increased feeling of boredom (Droit-Volet et al., 2020).
d) Additionally, in the context of a resource-limited country, other factors collide to increase stress and psychological burdening in the population. High rates of poverty, hunger, and delinquency, overcrowded households, had been reported to increase the risk of infection and negative outcomes in patients already infected (Shammi et al., 2020).

### Participants

Non-probability sampling was used for convenience. The target population was made up of adults, over 18 years old, of both sexes, who agreed to answer the form voluntarily online and were able to answer the questions. No previous screening of mental health conditions was conducted, and we were unable to suggest potential resources for free tele-mental health support since those were not a viable option in Peru during the time. The contact with the participants was asynchronous and at a single moment. According to the study’s aim and given the current situation, no exclusion criteria were considered since the pandemic has been affecting the general population regardless of any condition type or socio-cultural characteristic.

### Online Survey

We designed an online survey using Google Forms. It was shared via social media (i.e., WhatsApp and Facebook) using a snowball sampling. The survey was anonymized and volunteer; participants also had the opportunity to leave the survey at any moment if willing.

### Instruments

We constructed an online survey assessing psychological variables, and socio-demographic variables including age, sex, educational level, civil status, employment, exercise, and health status.

#### The Perceived Stress Scale

It is a self-reporting instrument assessing levels of perceived stress according to the thoughts and feelings of the last month (Cohen et al., 1983). It includes 10 items scored by a 5-point Likert scale with higher scores indicating higher levels of stress. The Perceived Stress Scale (PSS-10) consists of two dimensions: Perceived helplessness (6 items) and Perceived Self-Efficacy (4 items). Additionally, there is evidence of internal structure for the model of two correlated dimensions, invariance between men and women and, optimal internal consistency values (Liu et al., 2020). We defined moderate and severe stress symptoms using a cut-off of 14 and higher (Seedhom et al., 2019).

#### Positive Affect and Negative Affect Scale (PANAS)

This instrument is a 20-item scale that assesses mood with two factors, positive affect (PA) and negative affect (NA) (Watson et al., 1988). The two general or higher dimensions are the NA and the PA dimensions containing 10 items each. Higher scores on each of the subscales suggest a high presence of positive or negative emotions, using ordinal categories (Extremely, Fairly, Moderately, Slightly, Slightly, or Not at all). The test was self-administered.

#### Generalized Anxiety Disorder Scale

It is a self-report instrument that evaluates the physical and cognitive symptoms of anxiety over a 2-week period (Spitzer et al., 2006). This scale is one-dimensional, and it is composed of 7 items on a 4-point scale (0 = not at all; 3 = almost every day). The total score can vary from 0 to 21; likewise, its categories go from slight anxiety to severe anxiety (Bártolo et al., 2017). The GAD-7 was validated in the Peruvian context and presents evidence of a good fit for a one-dimensional model and has optimal internal consistency values (Zhong et al., 2015). We used a cut-off of 10 and above for moderate anxious symptoms (Plummer et al., 2016).

#### Patient Health Questionnaire

The Depression Module of the PHQ-9 is useful for the diagnosis of depressive disorders (Kroenke et al., 2001). It consists of 9 items on a 4-point scale (never on an almost daily). It evaluates the depressive symptomatology present in the last 2 weeks based on the criteria established in the DSM-5. The score can vary from 0 to 27, and the severity categories range from minimal to moderately severe and severe. The PHQ-9 has been validated in Peru, has a one-dimensional structure, and is invariant according to sex, age, and educational level. Also, it presents optimal levels of internal consistency (Villarreal-Zegarra et al., 2019). We used a cut-off of 10 and above to consider moderate depressive symptoms (Manea et al., 2015).

#### Fear of COVID-19

We included two independent questions to assess fear of COVID-19: (a) on a scale of 0–10, how much fear do you feel about getting infected? and (b) on a scale of 0–10, how much fear do you feel about infecting your family? Both questions were scored using a numerical rating scale (NRS). NRS had been used in similar previous studies and it has adequate psychometric properties while reporting valid and reliable information, when only numerical data is required without giving more qualitative detail (Fitzpatrick et al., 2020b; Lu et al., 2020).

### Data Analysis

First, the sociodemographic characteristics of the participants and the prevalence of mental health problems were analyzed. Additionally, we calculated the reliability coefficient alpha (α) of all the mental health questionnaires used. Second, was performed using the Spearman correlation coefficient between the variables of interest (fear of contagion, anxiety, NA, positive affection, and perceived stress) since it does not require a normal distribution. Cohort points were proposed for a small (*r_*s*_* > 0.20), moderate (*r_*s*_* > 0.50), and large (*r_*s*_* > 0.80) effect (Ferguson, n.d.). Third, two regression models were constructed to understand the factors that could explain both dimensions of perceived stress. More specifically, the first regression model only included controlled variables (sex, age, civil status, education level, work, exercise, and health status) to explain perceived helplessness and perceived self-efficacy. The second regression model (based on the first) added the independent variables of Fear of contagion, Fear of infecting others, anxious symptoms, depressive symptoms, NA, and PA. Finally, a structural regression model was performed with the independent variables. It was used as an estimator of robust maximum likelihood (Holtmann et al., 2016). The structural regression model was evaluated in two steps. The first step was to evaluate different goodness-of-fit indexes: root mean square error of approximation (RMSEA), standardized root mean-square (SRMR), comparative fit index (CFI), and Tucker Lewis Index (TLI). The cut-off points of CFI and TLI > 0.95, and RMSEA and SRMR < 0.08 were considered (Xia and Yang, 2019). The second step was to evaluate the amount of variance explained by perceived stress (output variables) through the coefficient of determination (*R*^2^). All analysis was performance in R Studio and STATA.

### Ethical Aspects

The study was approved by the ethics committee of Norbert Wiener University Ethics Committee (Exp. N° 104-2020). In addition, the study was anonymous and voluntary, so it does not pose a risk to participants.

## Results

Data were collected from 222 individuals. Twelve participants were removed from the database, for presenting Mahalanobis distance values that exceeded the critical acceptable value, being considered multivariate outliers. Finally, the sample was composed of 210 participants. The age range was from 15 to 74 years, of which 39% (*n* = 82) were women. A 66.2% of the participants had a university education and 43.3% came from a nuclear family. Additionally, 65.2% of the participants had symptoms of at least one of the mental health conditions studied (*n* = 137; depression, anxiety or stress symptoms). The summary of the sociodemographic characteristics of the sample is found in Table 1.

**TABLE 1:** Sociodemographic characteristics of the participants (*n* = 210).

Variable	Categories	*n* (%)
Sex	Woman	128 (61.0)
	Male	82 (39.0)
Civil status	Single	156 (74.3)
	Married/cohabiting	40 (19.0)
	Separated/divorced	14 (6.7)
Educational level	Elementary/high school	25 (11.9)
	Technical education	30 (14.3)
	University education	139 (66.2)
	Graduate education	16 (7.6)
Work	Employed	143 (68.1)
	Unemployed	67 (31.9)
Exercise	Yes	116 (55.2)
	No	94 (44.8)
Health status	Self-reported disease	48 (22.9)
	Healthy	162 (77.1)
Anxiety	Yes	38 (18.1)
	No	172 (81.9)
Depression	Yes	41 (19.5)
	No	169 (80.5)
Stress	Yes	135 (64.3)
	No	75 (35.7)

*Exercise was defined as: Do you do intense physical activity at least once a week? depression was defined as a scored of 10 or higher in the PHQ-9 test; anxiety was defined as a scored of 10 or higher in the GAD-7 test, stress was defined as a score 14 or higher in the PSS-10 test.*

The reliability coefficients of all the mental health questionnaires were appropriate. We identified that the dimensions of perceived stress are moderately related to other variables. Perceived self-efficacy and positive affection are related (*r_*s*_* = 0.57); while perceived helplessness is related to anxious symptoms (*r_*s*_* = 0.64) and NA (*r_*s*_* = 0.69). Furthermore, both dimensions of perceived stress are identified as being related to each other (*r_*s*_* = 0.57). In Table 2 the correlations between all the variables used can be identified, all the correlation values were significant (*p* < 0.05).

**TABLE 2:** Spearman correlation analysis (*n* = 210).

	*M*	SD	(1)	(2)	(3)	(4)	(5)	(6)	(7)	(8)	α
1. Fear of contagion	5.7	2.79	1.00								–
2. Fear of infecting others	7.66	2.87	0.58*	1.00							–
3. Anxious symptoms	12.5	4.4	0.36	0.34	1.00						0.88
4. Negative affect	18.3	6.52	0.25	0.28	0.64*	1.00					0.90
5. Positive affect	26.6	8.82	–0.03	–0.02	–0.16	0.00	1.00				0.93
6. Perceived helplessness	15.1	5.62	0.29	0.29	0.64*	0.69*	–0.06	1.00			0.89
7. Perceived self-efficacy	12.9	3.77	–0.07	–0.06	–0.26	–0.23	0.57*	–0.04	1.00		0.85
8. Depressive symptoms	14.8	5.2	0.27	0.26	0.71*	0.66*	–0.22	0.60*	–0.24	1.00	0.90

*M*, mean; SD, standard deviation; α, reliability coefficient alpha; *moderate correlation. Fear of contagion by Numeric Rating Scale (NRS) of the question: How afraid are you of getting infected with COVID? Fear of infecting by NRS of the question: How afraid are you of infecting others? Anxiety symptoms by 7-item Generalized Anxiety Disorder (GAD-7); negative and positive affect measured by the Positive Affect and Negative Affect Scale (PANAS); perceived helplessness and Perceived self-efficacy were measured by the 10-item Perceived Stress Scale (PSS-10). Depressive symptoms measured by the Patient Health Questionnaire (PHQ-9).

Regression models showed that sex (β = −1.64; *p* = 0.001), anxiety symptoms (β = 0.38; *p* < 0.001), and NA (β = 0.33; *p* < 0.001) were the variables that most explained the perceived helplessness. While PA (β = 0.23; *p* < 0.001) and NA (β = −0.12; *p* = 0.012) were the variables that most explained the perceived self-efficacy (see Table 3).

**TABLE 3:** Lineal regression models that explain the dimensions of perceived stress (*n* = 210).

	Perceived helplessness		Perceived self-efficacy	
	Model 1^*a*^	Model 2^*b*^	Model 1^*a*^	Model 2^*b*^
	β (95% CI)	β (95% CI)	β (95% CI)	β (95% CI)
**Sex**				
Woman	Ref.	Ref.	Ref.	Ref.
Male	–2.41 (–3.85 to –0.97)***	–1.64 (–2.71 to –0.58)**	0.93 (–0.08 to 1.94)	0.04 (–0.84 to 0.93)
**Age**	–0.06 (–0.14 to 0.01)	–0.01 (–0.06 to 0.05)	0.04 (–0.01 to 0.09)	0.02 (–0.02 to 0.07)
**Civil status**				
Single	Ref.	Ref.	Ref.	Ref.
Married/cohabiting	–1.01 (–3.39 to 1.36)	–0.99 (–2.72 to 0.74)	0.05 (–1.62 to 1.71)	0.02 (–1.43 to 1.47)
Separated/divorced	–2.5 (–6.07 to 1.06)	–1.72 (–4.33 to 0.90)	–1.7 (–4.20 to 0.80)	–1.13 (–3.31 to 1.05)
Educational level	–0.38 (–1.31 to 0.56)	–0.15 (–0.85 to 0.56)	1.03 (0.38 to 1.69)**	0.46 (–0.13 to 1.05)
**Work**				
Employment	Ref.	Ref.	Ref.	Ref.
Unemployment	1.79 (0.20 to 3.38)*	0.29 (–0.91 to 1.50)	–1.18 (–2.29 to –0.06)*	–0.32 (–1.33 to 0.69)
**Exercise**				
Yes	Ref.	Ref.	Ref.	Ref.
No	0.18 (–1.27 to 1.64)	–0.49 (–1.55 to 0.58)	–0.48 (–1.5 to 0.54)	0.15 (–0.73 to 1.04)
**Health status**				
Healthy	Ref.	Ref.	Ref.	Ref.
Self-reported disease	2.62 (0.91 to 4.34)	0.64 (–0.62 to 1.91)	0.58 (–0.62 to 1.78)	0.64 (–0.42 to 1.7)
**Psychological variables**				
Fear of contagion		0.15 (–0.09 to 0.39)		–0.04 (–0.24 to 0.16)
Fear of infecting others		–0.09 (–0.34 to 0.15)		0.03 (–0.17 to 0.23)
Anxious symptoms		0.38 (0.19 to 0.58)***		–0.03 (–0.19 to 0.14)
Depressive symptoms		0.10 (–0.06 to 0.27)		0.05 (–0.09 to 0.19)
Positive affect		0.02 (–0.05 to 0.08)		0.23 (0.17 to 0.28)***
Negative affect		0.33 (0.21 to 0.44)***		–0.12 (–0.22 to –0.03)*
F-value (*p*-value)	6.29 (<0.001)	21.01 (<0.001)	3.50 (<0.001)	8.51 (<0.001)
*R*^2^ (adjusted *R*^2^)	0.20 (0.17)	0.60 (0.57)	0.12 (0.09)	0.38 (0.33)

*p < 0.05. **p < 0.01. ***p < 0.001. Values in bold are the variables of interest. ^*a*^Adjusted model by sex, age, civil status, education level, work, exercise, and health status. ^*b*^Adjusted model by sex, age, civil status, education level, work, exercise, health status, fear of contagion, fear of infecting others, anxious symptoms, depressive symptoms, negative affect, and positive affect. 95% CI = 95% confidence interval.

Model 2 was able to explain a greater amount of variance compared to model 1, both for perceived helplessness (57%) and perceived self-efficacy (33%). Therefore, the model that includes the sociodemographic and psychological variables (model 2) manages to explain more variability, compared to the model only of sociodemographic variables (model 1).

**FIGURE 2:**
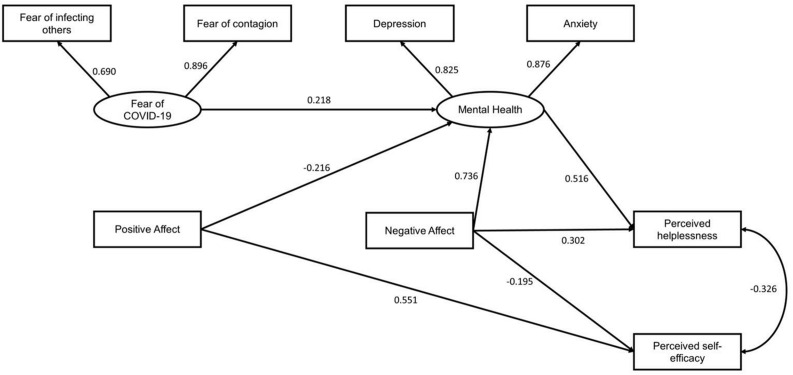
Explanatory model of dimensions of perceived stress. All coefficients presented were significant (*p* < 0.001).

The model presented have adequate indexes of the goodness-of-fit (*X*^2^ = 26.4; gl = 15; CFI = 0.983; TLI = 0.969; SRMR = 0.088; RMSEA[90% CI] = 0.063[0.017–0.101]). It is identified that fear of COVID-19 composed of fear of infecting others and fear of contagion, predicts the emergence of mental health problems (i.e., anxiety and depression symptoms); while mental health problems are predicted by PA and NA (see Figure 2). Perceived helplessness is predicted by mental health problems and NA. Perceived self-efficacy is predicted by NA and PA. Finally, Perceived helplessness and Perceived self-efficacy are related to each other, as they are part of the perceived stress variable. The model can predict 59% of the Perceived helplessness variance (*R*^2^ = 0.59) and 35% of the Perceived self-efficacy variance (*R*^2^ = 0.35).

## Discussion

### The Main Findings

Knowing the factors that explain perceived stress will allow us to understand one of the most important elements in the development of mental health problems, since stress is a nonspecific component that leads to more complex conditions such as anxiety, depression, and post-traumatic stress (Patel et al., 2018). Therefore, it is essential to understand how perceived stress is generated, since high prevalence of mental health problems have been reported during the pandemic (Wu et al., 2021). The present study proposed an exploratory model to identify the relevant factors associated with the perception of stress during the context of the COVID-19 pandemic. The seven hypotheses formulated in the proposed model were supported by the evidence presented. Specifically, higher levels of NA and mental health problems (i.e., anxiety and depression symptoms) explained perceived helplessness, while higher levels of PA and lower levels of NA explained perceived self-efficacy. While the fear of COVID-19, NA and PA were factors that explained the presence of mental health problems such as anxiety and depression.

### Comparison With Other Studies

Fear of contagion has been reported as a major stressor in unknown infectious outbreaks, especially during the context of pandemics (Fitzpatrick et al., 2020b). Therefore, it is justified that it is the variable that initiates the proposed model (see Figure 2). However, even though there is evidence between the relationship between fear and stress (Shin and Liberzon, 2010; Maeng and Milad, 2015), according to the proposed model, fear does not directly influence stress, but it is indirectly mediated by the presence of mental health problems, PA or NA.

Our study identified that fear of COVID-19, NA, and PA were factors that explained the presence of mental health problems such as anxiety and depression. Previous studies carried out during the context of the pandemic in the general population also identify a positive relationship between fear of COVID-19 and the presence of mental health problems such as anxiety, depression, and post-traumatic stress (Fitzpatrick et al., 2020a; Huarcaya-Victoria et al., 2020). Since fear is a precursor and a main trigger of the stress response (Onozuka and Yen, 2008), several longitudinal studies have identified that the presence of constant fear states can trigger emotional problems such as anxiety and depression (Lonigan et al., 2003).

Although in our study it was found that the relationship between fear of contagion and of being infected was small, it is plausible to consider that fear is the first step of a stress response, although by itself it would not explain the presence of perceived stress. On the other hand, other studies have already shown a positive relationship between NA and the presence of mental health problems such as anxiety and depression (Watson et al., 1988), a situation that has been exacerbated during the pandemic. Likewise, studies have been identified that find an inverse relationship between PA and the presence of these mental health problems (Everaert et al., 2020). A Spanish study proposes that PA and NA are mediators of anxiety, anger-hostility, depression, and joy (Pérez-Fuentes and Molero Jurado, 2020). This would imply a circular relationship between PA and NA with mental health problems, that is, if PAs increase, the levels of mental health problems will decrease, which implies a reduction in NA. The complexity of these relationships is beyond the scope of the study; however, it is important to be able to consider the circularity of these relationships for later studies.

Our study identifies that higher levels of NA and mental health problems (i.e., anxiety and depression) predict higher levels of perceived helplessness. Other studies have also identified a positive relationship between helplessness and the presence of depressive and anxious symptoms. A study carried out in victims of violence found that helplessness is related to the appearance of depressive symptoms (Salcioglu et al., 2017), while another study carried out in patients with myocardial infarction, found that learned hopelessness is related to the presence of depressive symptoms (Smallheer et al., 2018). In addition, during the COVID-19 context, an investigation carried out in the general population identified a positive relationship between NA and the presence of mental health problems such as anxiety and depression (Pérez-Fuentes and Molero Jurado, 2020). These investigations carried out before and during the pandemic support what was found in our study. On the other hand, negative affectivity has been identified as a common factor between anxiety, depression, and helplessness (Camuñas et al., 2019), so its position as a mediator between mental health problems and helplessness is logical. This justifies the approach presented in our study, where it is pointed out that NA mediates mental health problems and helplessness (Figure 2).

The present investigation reported that PA has a direct relationship with self-efficacy, while the latter is inversely related to NA. Other studies have identified this same relationship in people recovering from substance abuse, where it was identified that self-efficacy and NA have an inverse relationship (May et al., 2015). On the other hand, other studies carried out in patients with chronic diseases have found a positive relationship between PA and self-efficacy (Dunkley et al., 2017; Krok and Zarzycka, 2020; Smith et al., 2020). The available evidence suggests that self-efficacy increases the perception of having sufficient personal resources to cope with stressful situations, such as the context of the COVID-19 pandemic (Yıldırım and Güler, 2020).

### Public Health Implications

These findings represent a theoretical contribution to public health, under a critical analysis, these results allow reflection, providing a better understanding of the variables analyzed. Identifying fear and negative emotions as the main trigger for the development of mental disorders such as anxiety and depression proves the hypotheses raised and contributes to the existing literature.

The fear of COVID-19 throughout this period of pandemic has been characterized as being sustainable over time, it is no longer an acute reaction, in which the body responds in an adaptive way, to a stressful event, it is a chronic response, which is maintained over time, producing in the person an adaptation to damage and an allostatic load (Fofana et al., 2020; Raza et al., 2020). Consequently, fear being an emotion mediated by worrisome thoughts of uncertainty (Brosschot et al., 2006), threat or harm will generate emotional, cognitive, and behavioral consequences in the population, thus affecting not only mental health, but also health physical. Recognizing the importance of the role that emotions, whether positive or negative, play in population health will help decision makers and health workers to establish actions to promote the care and protection of mental health and reduce levels of perceived stress.

Likewise, understanding how perceived stress develops in its various forms of coping, during the context of the COVID-19 pandemic could serve as an indicator to promote preventive medicine as a public policy, and through it counteract a health reality affected by corruption, neglection and administrative inefficiency, which currently characterize health administration and management in the Peruvian population (García, 2019).

From these results, it is necessary to generate new study hypotheses, through longitudinal research proposals on the control of basic emotions, with quasi-experimental designs, to compare the efficacy of interventions, construction of instruments for the early detection of maladaptive behaviors in children and adolescents, validation of diagnostic programs and methods.

Finally, understanding that population health is comprehensive, prioritizing it will contribute to the reduction of poverty, optimizing the best conditions and quality of life for the population.

### Limitations and Strengths

Our study applies advance statistical methods using structural equation modeling, which allows for the analysis of different variables simultaneously. However, it is not free of limitations. First, a small sample of participants collected with a non-probabilistic strategy, so there may be difficulties in extrapolating the results to other contexts. Second, although we have data on perceived anxiety, depression, or stress, this does not substitute for clinical evaluations carried out by psychiatrists or psychologists, which could indicate if they have a clinical disorder. Third, at the time of data collection, we did not have validated instruments in our context on the fear of COVID-19, considering its usefulness in the analysis (Huarcaya-Victoria et al., 2020). Fourth, we conducted our survey via online. Several limitations had been associated with online surveys including not having a moderator for clarification or followed up question, increased sample bias which reduces representativeness, and difficulties for detecting response fraud (Ball, 2019). Also, the cross-sectional design prevents us from establishing causality, although the analysis proposes potential directions among the studied variables, these must be confirmed using longitudinal analysis.

## Conclusion

The present study proposed a model to understand perceived stress based on two correlated dimensions (self-efficacy and helplessness) in the Peruvian general population during the context of the COVID-19 pandemic. This exploratory model will allow for a better understanding of the role of fear of COVID-19, mental health problems, NA, and PA with the presence of perceived stress. Also, a high prevalence of mental health problems was identified, with an estimated 65.2% of participants having symptoms of at least one of the mental health conditions studied (depression, anxiety, or stress).

## Data Availability Statement

The datasets presented in this study can be found at Figshare (https://doi.org/10.6084/m9.figshare.14497923).

## Ethics Statement

The studies involving human participants were reviewed and approved by the Norbert Wiener University Ethics Committee. Written informed consent for participation was not required for this study in accordance with the national legislation and the institutional requirements.

## Author Contributions

AB-C: conceptualization, data gathering, descriptive data analysis, redaction, and supervision. AN-F: literature search, descriptive data analysis, manuscript writing, and English redaction formatting. DV-Z: conceptualization, regression data analysis, SEM analysis, redaction, figure drafting, and supervision. All authors contributed to the article and approved the submitted version.

## Conflict of Interest

The authors declare that the research was conducted in the absence of any commercial or financial relationships that could be construed as a potential conflict of interest.
